# Supplementary material for: Generating controlled gust perturbations using vortex rings
Source: PLoS One. 2024 Jul 8;19(7):e0305084. doi: 10.1371/journal.pone.0305084 (PMC11230548; doi:10.1371/journal.pone.0305084)
Supplement: S1 Appendix — (DOCX) [file pone.0305084.s003.docx]

# **APPENDIX**

## *Theoretical considerations for generating vortex rings*

*Estimation of slug length (L)*

Fluid is ejected out of the nozzle due to impulsive motion of the piston, the effective length of which is called slug length (*L*) (Fig. 1A). Assuming the fluid to be incompressible, the volume conservation (continuity) equation gives an estimate of the slug length in relation to the velocity profile and the duration of piston motion (Fig. 1B),

$\boldsymbol{L}\boldsymbol{=}\int\boldsymbol{U}_{\boldsymbol{s}}\boldsymbol{dt}\boldsymbol{=}\bar{\boldsymbol{U}_{\boldsymbol{s}}}\boldsymbol{T}_{\boldsymbol{p}}$ (1)

where $\bar{U_{s}}$ is the average velocity of the slug of fluid that leaves the nozzle exit. The slug length can thus be written as

$L=\frac{A_{p}\Delta X}{A_{0}}=\frac{D_{p}^{2}\Delta X}{D_{0}^{2}}$ (2)

where *A_p_* and *A_0_* are area of piston and nozzle respectively. Equations 1 and 2 essentially say that the volume of fluid emerging from the nozzle is equal to that pushed by the piston .

Alternatively, *L* can also be estimated by equating volume of fluid contained in the ring (without entrainment) with the volume of fluid swept by the piston [32], i.e.,

$\frac{\pi D_{r}^{3}e}{6}=\frac{\pi D_{o}^{2}L}{4}$ (3)

i.e., $\boldsymbol{L=}\frac{\boldsymbol{2}\boldsymbol{D}_{\boldsymbol{r}}^{\boldsymbol{3}}\boldsymbol{e}}{\boldsymbol{3}\boldsymbol{D}_{\boldsymbol{0}}^{\boldsymbol{2}}}$ (4)

Where *D_r_* is the diameter of the ring and *e* is its eccentricity (major axis diameter/minor axis diameter) (See Fig. 1E).

In the experiments reported here, D_r_ = 0.86D_vb_=7.3 cm, e =0.62, and D_0_ =3.7 cm. Inserting these values into equation 4,

$$L=\frac{2D_{r}^{3}e}{3D_{o}^{2}}=11.75 cm$$

And the formation number $L/D_{0}=3.17$

*Estimation of core radius (a)*

For cases where piston motion time is small, the thin vortex core assumption applies, and

the core radius (*a*) of the ring can be estimated [25]


 as:

$\boldsymbol{a=}\sqrt{\boldsymbol{4}\boldsymbol{\nu}\boldsymbol{T}_{\boldsymbol{p}}}$, where $\nu$ is kinematic viscosity of the fluid. (5)

*Estimation of translational velocity (*$U_{avg}$*)*

The velocity (*U_avg_*) of the fluid ejected out of the nozzle after it is fully formed into a ring can be estimated [25] as

$\boldsymbol{U}_{\boldsymbol{avg}}\boldsymbol{=}\frac{\boldsymbol{L}^{\boldsymbol{2}}}{\boldsymbol{4}\boldsymbol{\pi}\boldsymbol{D}_{\boldsymbol{r}}\boldsymbol{T}_{\boldsymbol{p}}}\boldsymbol{(ln}\frac{\boldsymbol{4}\boldsymbol{D}_{\boldsymbol{r}}}{\boldsymbol{a}}\boldsymbol{-0.558)}$ (6)

where *L^2^/2T_p_* gives the estimation of circulation of the slug.

Following momentum conservation, the momentum of the slug ejected out of nozzle can be equated to the momentum of the vortex bubble. This, however, holds only if the formation number ($L/D_{0}$) is approximately 4 [34].

$m_{s}\bar{U_{s}}=m_{vb}U_{avg}$ (7)

where *U_s_* is slug velocity, *m_s_* is the mass of fluid displaced by the piston, and *m_vb_* is the mass of the ring, which includes the entrained fluid (i.e. vortex bubble). The mass of the vortex bubble is given as $m_{vb}=m_{s}\left( 1+k \right)$, where *k*  is entrained mass fraction. The value of *k* can be determined from the value of eccentricity of the ellipsoid ring, *e*, which is the ratio of minor to major axis radii of the ellipsoid (see Fig. 1E) [32]. The entrained volume is about 20-40% total volume of the ring [27]. This implies that (7) can be written as

$\bar{\boldsymbol{U}_{\boldsymbol{s}}}=\boldsymbol{U}_{\boldsymbol{avg}}(\mathbf{1}+\boldsymbol{k})$. (8)

**List of symbols**

*a core radius of the vortex ring*

*A_p_ area of piston*

*A_0_ exit area of nozzle*

*D_n_ exit diameter of nozzle*

*D_p_ diameter of piston*

*D_r_ diameter of ring without entrainment*

*D_vb_ diameter of vortex bubble ( ring with entrained volume)*

*D_0_ exit diameter of nozzle*

*e eccentricity (= radius of minor axis/ radius of major axis)*

*f wing beat frequency*

*k entrained mass fraction (= entrained mass/mass of slug)*

*L slug length*

*m_s_ mass of slug coming out of nozzle*

*m_vb_ mass of vortex bubble*

*Re Reynolds number (=U­avg X D_0_/υ)*

*T no. of wing beat (=t*f)*

*T_n_ non-dimensional time of vortex ring(=tU_avg_/ D_0_)*

*T_p_ piston time*

*U_avg_ average propagation speed of vortex ring*

*U_n_ non-dimensional velocity*

*U_p_ piston velocity*

*U_s_ slug velocity*

*V_in_ input voltage*

*X_L_ axial distance non-dimensionalized by insect’s body length*

*X_n_ axial distance non-dimensionalized by exit diameter of nozzle*

*Z_L_ vertical distance non-dimensionalized by insect’s body length*

*∆X piston displacement*

*γ body roll angle (in °)*

*υ kinematic viscosity*

*CoM Center of Mass*

*FIP Fog Injection Port*
